# Congenital absence of the portal vein associated with focal nodular hyperplasia of the liver and congenital heart disease (Abernethy malformation): A case report and literature review

**DOI:** 10.3892/ol.2014.2767

**Published:** 2014-12-04

**Authors:** YABIN HAO, XU HONG, XINYAN ZHAO

**Affiliations:** 1 Department of Endocrinology, Beijing Friendship Hospital, Capital Medical University, Beijing 100050, P.R. China; 2 Department of Hepatology, Beijing Friendship Hospital, Capital Medical University, Beijing 100050, P.R. China

**Keywords:** focal nodular hyperplasia, ventricular septal defect, portal vein, portosystemic shunt

## Abstract

Abernethy malformation is a rare congenital malformation defined by an extrahepatic portosystemic shunt. The majority of affected patients are young (<18 years of age) and experience various symptoms, including vomiting, jaundice, dyspnea and coma. The current study presents a case of Abernethy malformation in an asymptomatic adult male patient. The patient exhibited congenital absence of the portal vein, congenital heart disease (postoperative ventricular septal defect status), and multiple liver lesions, confirmed to be focal nodular hyperplasia by biopsy. Ultrasonography and magnetic resonance imaging findings revealing the liver lesions, type II congenital absence of the portal vein and the portosystemic shunt are presented. In addition, the common clinical presentations, associated anomalies, diagnostic workup and treatment options of this disorder are investigated by reviewing 101 previously reported cases.

## Introduction

Abernethy malformation is an extremely rare congenital vascular malformation that is characterized by the diversion of portal blood away from the liver (1). It frequently comprises a number of congenital anomalies of the liver, including congenital absence of the portal vein (CAPV), portosystemic shunt, and liver nodules, as well as congenital heart diseases such as atrial septal defect, ventricular septal defect (VSD) and patent ductus arteriosus (2).

The number of CAPV diagnoses has increased in recent years due to advances in imaging techniques (3,4). To the best of our knowledge, 101 cases of CAVP have been reported since the condition was first described in 1793 (1), and the majority of affected patients were <18 years of age and female (1,3–90). Almost all adult patients in the reported cases that have been reviewed in the present study were admitted to hospital with various symptoms including nausea, vomiting, fatigue, epigastric pain, asthenia, anorexia, jaundice and dyspnea (3–31). In general, the treatment options for CAPV include surgical correction of shunts, liver nodule resectioning and liver transplantation. The outcome of CAPV in adults is good, and may be improved further by adopting appropriate treatment strategies.

The current study reports the case of multiple focal nodular hyperplasia (FNH) with CAPV in an asymptomatic adult male patient of postoperative VSD status. Written informed consent was obtained from the patient.

## Case report

A 19-year-old male was admitted to the Endocrinology Department, Beijing Friendship Hospital (Beijing, China), due to a mildly elevated alanine transaminase level that had been incidentally identified during a routine health examination. No clinical evidence of encephalopathy or weight loss was observed. Physical examination revealed no abnormalities with the exception of mild jaundice and a diastolic murmur at the upper left sternal border. The patient’s medical history included VSD and pulmonary valve stenosis, and the patient had undergone surgical VSD repair at 3 years of age. The mother also reported a mild viral infection (a cold) in her early pregnancy, which may have affected early fetal development.

Liver function testing showed an alanine transaminase level of 51 U/l (normal, 0–40 U/l), a γ-glutamyl transpeptidase level of 134 U/l (normal, 8–55 U/l), a total bilirubin level of 52.46 μmol/l (normal, 3.42–17.1 μmol/l) and an indirect bilirubin level of 36.83 μmol/l (normal, 0–12 μmol/l). Routine blood tests and a reticulocyte analysis revealed a white blood cell count of 6.8×10^9^/l (normal range, 4.0–10.0×10^9^/l), a neutrophil level of 3.6×10^9^/l (normal range, 2.0–7.0×10^9^/l), a red blood cell count of 5.5×10^12^/l (normal range, 4.0–5.5×10^12^/l), a hemoglobin level of 167 g/l (normal range, 120–160 g/l), platelet level of 226×10^9^/l (normal range, 100–300×10^9^/l) and reticulocyte level of 0.02×10^12^/l (normal range, 0.01–0.09×10^12^/l). No fragmented red blood cells were identified in the peripheral blood. The coagulation profile was normal. Serological markers for ceruloplasmin and hepatitis A–E viruses as well as immunological markers were negative; α-fetoprotein was also negative.

**Figure 1:**
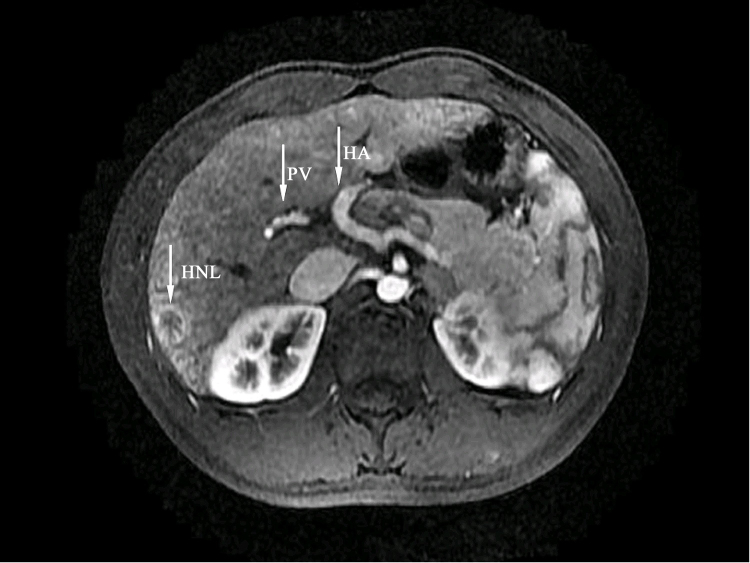
Abdominal magnetic resonance image showing the slender portal vein, rich arterial blood supply and hepatic mass lesion. HA, hepatic artery; PV, portal vein; HNL, hepatic nodular lesion.

Abdominal ultrasonography revealed a number of hypoechoic solid masses distributed in the right and left lobes of the liver, and an enlarged spleen. A splenorenal shunt was also detected. Magnetic resonance imaging was advised based on the abdominal ultrasound findings (Fig. 1). Magnetic resonance imaging confirmed the presence of a splenorenal shunt and multiple lesions with rich blood supplies. It also showed that the portal vein was slender with unclear branching. These findings led to the diagnosis of type II CAPV.

**Figure 2:**
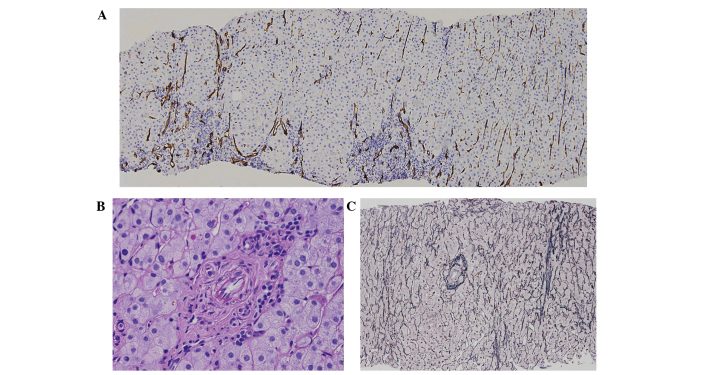
(A) Immunohistochemical staining for CD34 in biopsy specimen from the liver nodule showed hyperplasia of arterioles. (B) Periodic acid-Schiff diastase staining revealed thick-walled arterioles. (C) Reticular silver staining showed hydropic degeneration of hepatocytes; fibrosis in the portal area and was confirmed to be a focal nodular hyperplasia.

Percutaneous fine-needle aspiration biopsy of the liver nodules was subsequently performed (Fig. 2). Pathological examination revealed bile duct proliferation, hydropic degeneration of hepatocytes, hyperplasia of thick-walled arterioles and fibrosis in the portal area, but no intrahepatic cholestasis. The lesions were therefore confirmed to be FNH.

Electrocardiography revealed a right bundle branch block, and ultrasonic cardiography indicated congenital heart disease, postoperative VSD status, pulmonary valve stenosis and pulmonary regurgitation.

## Discussion

The term CAPV was first coined by John Abernethy in 1793. Congenital extrahepatic portosystemic shunt is also known as Abernethy malformation in recognition of its initial identification (1). Complete portosystemic shunts that do not perfuse the liver via the portal vein are defined as type I, whereas partial shunts with a remaining degree of portal perfusion to the liver are defined as type II. Type I is further subclassified into types Ia and Ib according to the course of the splenic and mesenteric veins (32).

A number of patients with Abernethy malformation have been previously described (1,3–89). Among them, 66 were female and 35 were male, with ages ranging from fetus to 61 years at the time of diagnosis. In total, 70 patients (69.30%) were<18 years of age, and <10% had type II malformations (45,58,75–77).

In the present case, imaging findings indicated that the portal vein had formed by the union of the splenic and superior mesenteric veins. These veins were present, but appeared slender and hypoperfused as a portion of the blood was being diverted into the inferior vena cava via a splenorenal shunt. Therefore, a diagnosis of type II Abernethy malformation was determined.

In addition to the absence of the portal vein, nodular liver lesions were observed in almost half of the reported cases (48.51%). The association between portal vein agenesis and nodular liver lesions is attributed to the absence of portal blood flow and compensatory increased hepatic arterial blood flow. Systemic shunting of the visceral venous return may lead to abnormal development, malfunction and regeneration of the liver, secondary to the absence of portal hepatotrophic factors, resulting in the development of hepatic lesions (87). The majority of these lesions were characterized as benign, such as FNH (36.73%). Other reported lesions included nodular regenerated hyperplasia (16.33%), hepatoblastoma (4.08%), hepatic adenoma (10.20%), hepatocellular carcinoma (26.53%) and cirrhosis (6.12%). In the current review, only a few patients with CAPV associated with FNH were male (8.16%; Table I).

The patient in the present case was in reasonable health following heart surgery and visited a doctor for the evaluation of an abnormal result obtained during a regular health examination. Although the patient felt no discomfort, the ultrasonography report indicated a more serious condition. Following a series of imaging examinations and a biopsy, malignant lesions were ruled out and it was concluded that the characteristic hepatic changes were secondary to the congenital malformation of the portal vein, and were associated with congenital heart disease.

Several known associations between primary liver disease and concomitant congenital cardiac defects have been identified (2). Congenital cardiac diseases including atrial septal defect, patent foramen ovale, VSD and patent ductus arteriosus are frequently observed concurrent with CAPV. Congenital stenosis of the aortic valve and pulmonary artery valve, observed in a number of patients with CAPV, can cause tricuspid regurgitation (36,44,90). However, it has also been hypothesized that systemic shunting of portal venous flow could adversely affect hepatic and cardiac development and function. Concomitant atrial and ventricular septal defects associated with CAPV may be attributed to a congenital adaptive change that occurs during development from the embryonic stage, which tends to compensate for the congestive effects of portal venous aplasia (2).

Another possible cause of vascular dysplasia is viral infection in early pregnancy, as occurred in the present case, where the patient’s mother reported a mild viral infection (a cold) during early pregancy. Embryologically, the portal vein originates from the paired vitelline veins. Between gestational weeks four and five, the paired vitelline veins form three anastomoses that, over the course of the first trimester, undergo selective involution to produce the portal vein (91). Aberrations in this process of involution may result in anatomical variations within the portal system; specifically, excessive involution may result in the absence of the portal vein. Almost simultaneously, a wall forms, separating the right and left ventricles. If the wall does not completely form, a hole remains. This hole is known as a VSD. Any abnormality in this process of involution may lead to VSD.

Among the reported cases, 46 cases associated with a variety of congenital anomalies were identified (92). In addition to the aforementioned congenital cardiac disease (16/46; 34.78%), other types of dysplasia involved the kidney (6/46; 13.04%), spleen (5/46; 10.87%), bone (4/46; 8.70%), arteries (3/46; 6.52%), bile duct (3/46; 6.52%), nervous system (2/46; 4.35%), urethra (1/46; 2.17%) and endocrine glands (1/46; 2.17%). In addition, a number of patients were affected by Turner syndrome (2/46; 4.35%), Caroli syndrome (1/46; 2.17%), Goldenhar syndrome (1/46; 2.17%) and Down syndrome (1/46; 2.17%; Table I).

The type of CAPV and simultaneous presence of congenital anomalies are the key factors in determining the severity of a given patient’s pathogenetic condition and the course of the disease. Furthermore, the mode of management should be established on a case-by-case basis, according to the type or anatomy of the disease, in addition to the symptoms and clinical condition of the patient.

Treatments may include liver transplantation, balloon-occluded retrograde transvenous obliteration, embolization with metallic coils and surgical correction of shunts (93). In patients with type II malformations previously diagnosed with CAPV, occlusion of the shunt is indicated in cases with serious symptoms such as hepatic encephalopathy (43) or lateral bleeding. In the present case, the patient was asymptomatic, and the physical examination findings and laboratory test results appeared normal. Thus, we propose that close clinical, biochemical, and imaging follow-up must be performed and that interventional treatment should not be immediately conducted.

In conclusion, the diagnosis of Abernethy malformation and its associated anomalies is challenging. It is important not only to detect portal vein malformation, but also to identify other important associated anomalies, due to the variable clinical consequences. It appears that the long-term prognosis hinges on adequate control of the hepatic dysfunction and metabolic derangements; however, only longitudinal follow-up of these patients will provide further insight.

## Figures and Tables

**Table I:** Congenital absence of the portal vein, abernethy malformation: Summary of reported cases.

	Male		Female	
		
Group	≥18	<18	≥18	<18
Case numbers, n	9	26	22	44
Type: I/II/NA, n	7/2/0	15/3/8	7/2/13	11/2/31
FNH/NRH, n	2/1	2/2	7/2	7/3
Adenoma, n	0	0	3	2
HCC, n	2	4	2	5
Hepatoblastoma, n	0	0	0	2
Cirrhosis, n	0	1	1	1
CHD, n	2	6	2	6
Kidney dysplasia, n	1	2	1	2
Spleen dysplasia, n	0	1	2	2
Dysostosis, n	0	2	0	2
Arteries malformation, n	0	1	1	1
Bile duct dysplasia, n	0	0	0	3
Other coexistence congenital anomalies	0	Urethra,1 Nervous system,1	Caroli syndrome,1	Endocrine gland,1 Nervous system,1 Turner syndrome,2 Goldenhar syndrome,1 Down syndrome,1

NA, not applicable (not described in the reports); FNH, focal nodular hyperplasia; NRH, nodular regenerated hyperplasia; HCC, hepatocellular carcinoma; CHD, congenital heart disease.
